# Spectroscopic Properties of Pr^3+^, Tm^3+^, and Ho^3+^ in Germanate-Based Glass Systems Modified by TiO_2_

**DOI:** 10.3390/ma16010061

**Published:** 2022-12-21

**Authors:** Marta Kuwik, Karolina Kowalska, Joanna Pisarska, Wojciech A. Pisarski

**Affiliations:** Institute of Chemistry, University of Silesia, Szkolna 9, 40-007 Katowice, Poland

**Keywords:** glass systems, lanthanide ions, luminescence properties, CIE chromaticity coordinates, visible light emitters

## Abstract

In this paper, the effect of the GeO_2_:TiO_2_ molar ratio in glass composition on the spectroscopic properties of germanate glasses was systematically investigated. The visible luminescence bands associated with characteristic ^1^D_2_ → ^3^H_4_ (red), ^5^S_2_, ^5^F_4_ → ^5^I_8_ (green), and ^1^D_2_ → ^3^F_4_ (blue) transitions of Pr^3+^, Ho^3+^, and Tm^3+^ ions in systems modified by TiO_2_ were well observed, respectively. It was found that the luminescence intensity of glasses containing Pr^3+^ and Ho^3+^ ions increases, whereas, for Tm^3+^-doped systems, luminescence quenching with increasing content of TiO_2_ was observed. Based on Commission Internationale de I’Eclairage (CIE) chromaticity coordinates (x, y) analysis, it was demonstrated that the value of chromaticity coordinates for all glasses depends on the GeO_2_:TiO_2_ molar ratio. The addition of TiO_2_ to system compositions doped with Tm^3+^ ions shifts the (x, y) to the center of the CIE diagram. However, chromaticity coordinates evaluated for glasses containing Pr^3+^ ions move to a purer red color. Our results confirm that the spectroscopic properties of the studied glasses strongly depend on TiO_2_ content. Moreover, it can be stated that germanate-based glass systems modified by TiO_2_ can be used for optoelectronics in RGB technology as red (Pr^3+^), green (Ho^3+^), and blue (Tm^3+^) emitters.

## 1. Introduction

Over the past years, the progress in the capacity of modern optoelectronic and photonic technologies has contributed to the increasing interest in luminescence materials, particularly glasses, glass ceramics, and ceramics [1,2,3,4,5,6]. Inorganic systems doped with trivalent lanthanide (Ln^3+^) ions have been paid attention due to their spectroscopic properties in visible and near-infrared spectral range and possible applications as luminescent solar converters [7], solid-state lighting [8], generators of white light [9], optical thermometers [10,11], laser and broadband fiber amplifiers [12,13]. One of the more interesting uses of glasses doped with Ln^3+^ ions is the production of emitters for RGB technology [14]. In particular, systems containing rare earth ions such as praseodymium ions (Pr^3+^) are promising red-emitting materials [15,16]. Sun et al. [17] reported that Pr^3+^-doped aluminosilicate glasses may be a potential candidate for efficient visible fiber lasers operated at 610 and 640 nm wavelengths. Moreover, glasses doped with holmium ions (Ho^3+^) are being extensively studied due to possible green luminescence [18,19]. As Rani et al. [20] suggested, relatively higher values of stimulated emission cross-section and quantum efficiency observed for ^5^S_2_ → ^5^I_8_ transition allow us to contemplate that Ho^3+^-doped borate glass systems can be used for fabricating green lasers. On the other hand, materials containing thulium ions (Tm^3+^) due to characteristic luminescence properties are analyzed in the visible blue region [21,22,23].

It is worth noting that the luminescence properties of Ln^3+^ ions are sensitive to the chemical composition of the host matrix. The presence of modifier oxides has an equally significant influence on the structural, thermal, and spectroscopic properties of materials doped with Ln^3+^ ions. Particularly noteworthy are metal oxides such as titanium (IV) oxide (TiO_2_), which is a useful component of the glass and glass ceramics systems [24,25,26,27]. The addition of TiO_2_ to the glass matrix becomes very interesting for developing new optical materials due to good physical, chemical, electrical, and optical properties and high transparency in the visible and near-infrared (NIR) regions [28]. Furthermore, modifier oxide TiO_2_ increases the linear and non-linear refractive indices, increasing the radiative transition probabilities and improving the non-linear optical properties [29]. Thermal studies indicate that glasses with low titanium (IV) oxide content exhibit high stability against crystallization [30]. The presence of small quantities of TiO_2_ in glass matrices is observed to enhance the glass-forming ability and chemical durability of the glasses [31]. However, the previously published results indicated that the oxide modifier TiO_2_ added to some host matrices effectively promotes glass crystallization, and it is quite difficult to synthesize thermally stable and fully amorphous systems with a high TiO_2_ concentration [32,33,34]. To the best of our knowledge, the spectroscopic properties of rare earth ions in germanate-based glass matrices are not frequently examined in the function of high concentrations of titanium (IV) oxide.

Keeping in mind the above considerations, in this paper, spectroscopic properties of Pr^3+^, Ho^3+^, and Tm^3+^ have been investigated in GeO_2_/TiO_2_-based glass systems. Spectroscopic properties of selected rare earth ions were analyzed as a function of the molar ratio GeO_2_:TiO_2_ (from 5:1 to 1:5) in the glass composition. The excitation and visible emission spectra for germanate glass systems were registered and discussed. Based on measurements, the Commission Internationale de I’Eclairage (CIE) chromaticity coordinates (x, y) for all glasses depending on the content of GeO_2_ and TiO_2_ were defined.

## 2. Materials and Methods

A series of multicomponent oxide glasses containing various molar ratio GeO_2_:TiO_2_ and singly doped with praseodymium (Pr^3+^), thulium (Tm^3+^), and holmium (Ho^3+^) ions were synthesized by a conventional melt-quenching method. The following nominal chemical composition (60-x)GeO_2_-xTiO_2_-30BaO-(10-y)Ga_2_O_3_-yLn_2_O_3_ (Ln = Pr, Tm, Ho; x = 10, 20, 30, 40, 45, 50; y = 0.1) of systems are given in molar%. In the present study, the appropriate metal oxides of high purity (99.99%, Aldrich Chemical Co. St. Louis, MO, USA) were weighted and mixed in an agate mortar. After homogenization, each batch of 5g was melted in a non-covered corundum crucible (Łukasiewicz Research Network, Institute of Ceramics and Building Materials, Cracow, Poland) in a high-temperature electrical furnace (FCF 4/170M produced by Czylok, Jastrzębie Zdrój, Poland) at 1250 °C in ambient air. The glass samples were kept at this temperature for 60 min before slowly being cooled down to room temperature. Finally, glass samples were shaped and polished to meet the requirements for photoluminescence spectral measurements.

Optical spectroscopy was used to examine the influence of the GeO_2_:TiO_2_ molar ratio on the luminescence properties of the studied glass systems. The excitation and emission spectra were recorded using a Photon Technology International (PTI) Quanta-Master 40 (QM40) UV/VIS Steady State Spectrofluorometer (Photon Technology International, Birmingham, NJ, USA) coupled with a tunable pulsed optical parametric oscillator, pumped by the third harmonic of a Nd:YAG laser (Opotek Opolette 355 LD, OPOTEK, Carlsband, CA, USA). The laser system was equipped with a double 200 mm monochromator, a xenon lamp (75W) (USHIO Inc. Japan) as a light source, and a multimode UVVIS PMT (R928) (PTI Model 914) detector (Photon Technology International, Birmingham, NJ, USA). All measurements were carried out at room temperature.

## 3. Results and Discussion

### 3.1. Characterization of Germanate-Based Glass Systems Modified by TiO_2_

For the germanate-based glasses to be employed for useful application in RGB technology, their structural and thermal characterization is essential. Therefore, in the first step of characterization of properties of germanate-based glass systems, the phase analysis was conducted using X-ray diffraction (XRD). Independently of TiO_2_ concentration, it was found that the X-ray diffraction patterns show two broad peaks characteristic of an amorphous state. Hence, it has been confirmed that the obtained germanate glasses are fully amorphous. It is interesting to notice that the glass-forming region for the studied compositions is relatively broad. Our previous results indicate that the type of rare earth ions does not affect the crystallization of the local structure of germanate systems modified by TiO_2_ [35].

Moreover, the glass transition phenomenon and, hence, the amorphous phase can be studied from the analysis of glass transition temperature (T_g_). This parameter is one of the most fundamental material factors, and it has a critical impact on the suitability of a system for optoelectronic applications. There are a variety of thermal and mechanical analytical techniques that can be used to measure this crucial parameter. In our study, we used Differential Scanning Calorimetry (DSC) to characterize the thermal properties of germanate-based glasses. Details are given in Ref. [36]. The obtained results show that the value of T_g_ increases in the presence of TiO_2_ in the glass host. When the glass transition of a system is situated as a function of the composition, the change in T_g_ values indicates definite changes in the glass network. Based on the thermal characterization, the studied systems suggested less open glass structure for germanate samples modified by TiO_2_. Additionally, it was found that the thermal stability parameter (ΔT) is reduced for the glass systems, where the concentration of TiO_2_ increases. However, this important factor is still above 100 °C, exhibiting the good thermal stability of germanate-based glasses.

Another critical parameter is the phonon energy of the glass host (hν), which can be determined based on excitation spectra [37]. The value of phonon energy reveals essential information regarding the modification of glass structure with a change in the composition. Our studies indicate that the phonon energy of the glass host is reduced for systems modified by TiO_2_ [36]. Previously published results demonstrate that germanate glasses are perspective materials as oxide glass host matrices for rare earth ions thanks to their favorable properties, such as smaller multiphonon relaxation probabilities due to relatively low phonon energy [38,39,40]. It can be assumed that the presence of TiO_2_ in glass composition causes the reduction in matrix phonons and improves the efficiency of the visible luminescence of praseodymium, holmium, and thulium ions in germanate-based glass systems. For that reason, the visible luminescence of Pr^3+^, Ho^3+^, and Tm^3+^ ions in the obtained glass samples are presented and discussed in detail in the next sections.

### 3.2. Spectroscopic Properties of Ln^3+^-Doped Germanate-Based Glass Systems

The influence of chemical composition on the spectroscopic properties of glass systems singly doped with trivalent praseodymium, holmium, and thulium ions is a key issue, confirmed by previous investigations [41,42,43,44]. Therefore, in our study, the excitation and emission spectra in the visible range for the studied glasses were registered in the function of the GeO_2_:TiO_2_ molar ratio.

**Figure 1:**
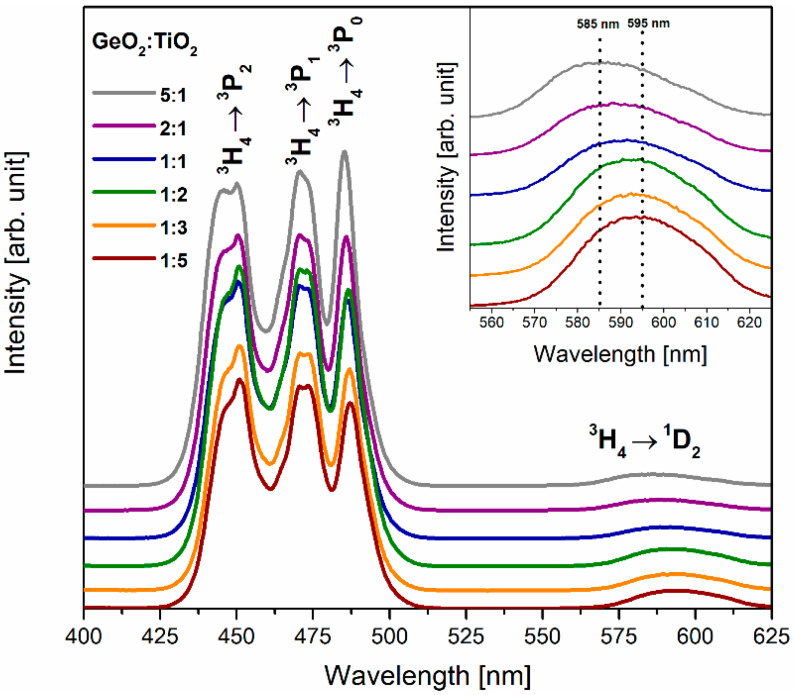
Excitation spectra for Pr^3+^ ions in TiO_2_-modified germanate glasses. The inset presents a shift of the maximum of excitation band due to ^3^H_4_ → ^1^D_2_ transition.

Firstly, the excitation spectra of TiO_2_-modified germanate glass systems doped with praseodymium ions were monitored at λ_em_ = 645 nm. All registered spectra show four bands assigned to transition originating from the ^3^H_4_ ground state to higher-lying ^3^P_2_, ^3^P_1_, ^3^P_0_, and ^1^D_2_ states (Figure 1). The most intense bands centered at 450 nm (^3^H_4_ → ^3^P_2_), 471 nm (^3^H_4_ → ^3^P_1_), and 486 nm (^3^H_4_ → ^3^P_0_) overlap, giving a characteristic profile. The energy gaps between excited states ^3^P_2_, ^3^P_1_, ^3^P_0_ are very small, and the excitation energy transfers very fast from the ^3^P_2_ state via ^3^P_1_ to the 3P_0_ state by non-radiative relaxation. Next, the ^3^P_0_ excited state is depopulated, giving several radiative transitions to the lower-lying states of Pr^3+^ ions. The band corresponding to the transition from the ^3^H_4_ ground state to the excited state ^1^D_2_ was also observed. It is worth noting that the maximum of this band is shifted to a longer wavelength region, and intensity increases with the change of GeO_2_:TiO_2_ molar ratio (Inset of Figure 1).

**Figure 2:**
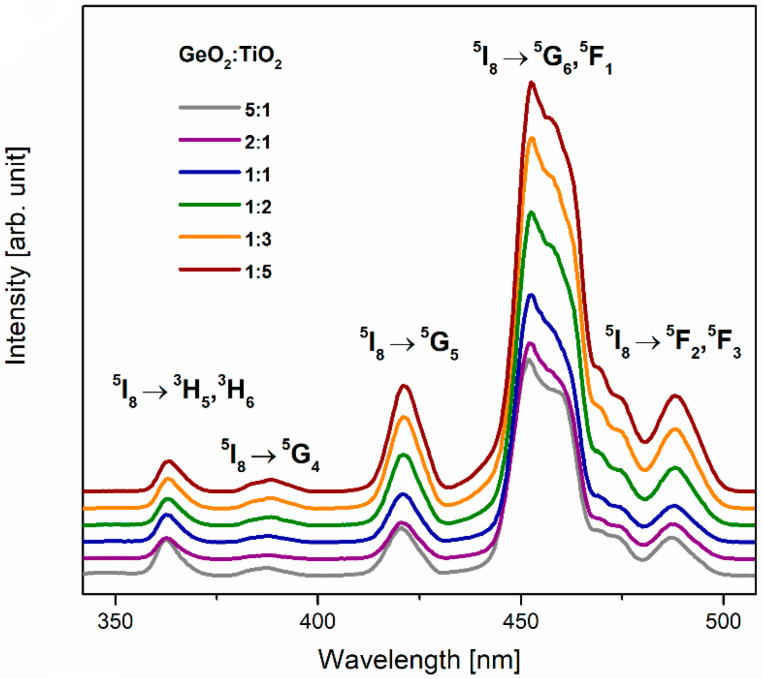
Excitation spectra for Ho^3+^ in TiO_2_-modified germanate glasses.

Figure 2 presents spectra (monitored at λ_em_ = 547 nm) of TiO_2_-modified germanate glass systems with five excitation bands corresponding to the transition from the ^5^I_8_ ground state of Ho^3+^. The observed bands of relatively low intensity located in the ultraviolet range (363 nm and 389 nm) can be attributed to ^5^I_8_ → ^3^H_5_, ^3^H_6_ and ^5^I_8_ → ^5^G_4_ transitions. However, the most intense excitation bands were measured in the wavelength range of visible radiation and centered at 450 nm (^5^I_8_ → ^5^G_6_, ^5^F_1_). It should be stated that the intensities of all excitation bands depend on GeO_2_:TiO_2_ molar ratios. Moreover, the intensities of bands located at 389, 420, 450, and 488 increased with a change of glass composition, whereas the decrease in intensity was observed well for the excitation band associated with the ^5^I_8_ → ^3^H_5_, ^3^H_6_ transition of Ho^3+^ ions.

**Figure 3:**
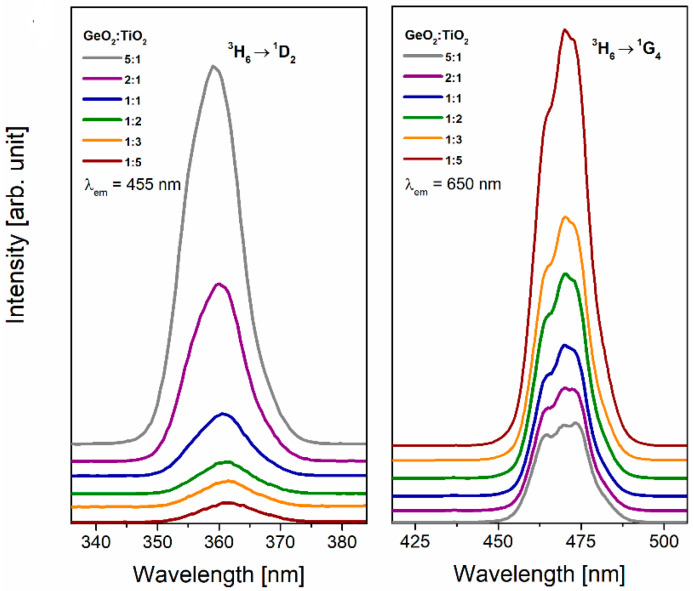
Excitation spectra for Tm^3+^ in TiO_2_-modified germanate glasses.

The excitation spectra for germanate-based glass systems were registered and monitored at 455 nm and 650 nm, the wavelengths corresponding to blue (^1^D_2_ → ^3^F_4_) and red (^1^G_4_ → ^3^F_4_) emissions of thulium ions (Figure 3). Independently on the GeO_2_:TiO_2_ molar ratio in the glass composition, only two bands corresponding to the transition from the 3H6 ground state to the excited states ^1^D_2_ (λ_em_ = 455 nm) and ^1^G_4_ (λ_em_ = 650 nm) were observed. It is interesting that the intensity of excitation bands strongly depends on the concentration of glass formers. The intensity of band originating from the ^3^H_6_ → ^1^G_4_ transition (λ_em_ = 650 nm) significantly decreases with the change in the GeO_2_:TiO_2_ molar ratio. On the other hand, the increase in the intensity of the second excitation band ^3^H_6_ → ^1^D_2_ (λ_em_ = 455 nm) was observed.

**Figure 4:**
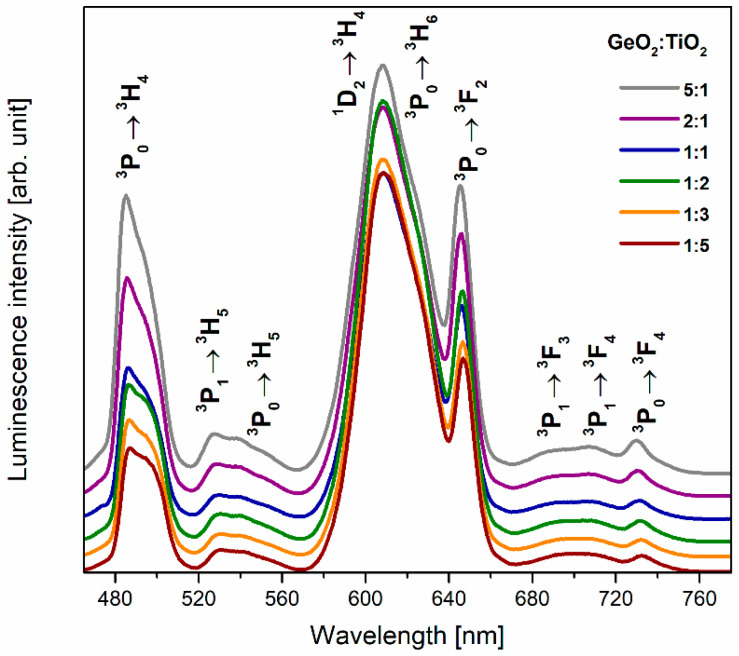
Luminescence spectra for Pr^3+^ ions in TiO_2_-modified germanate glasses.

**Figure 5:**
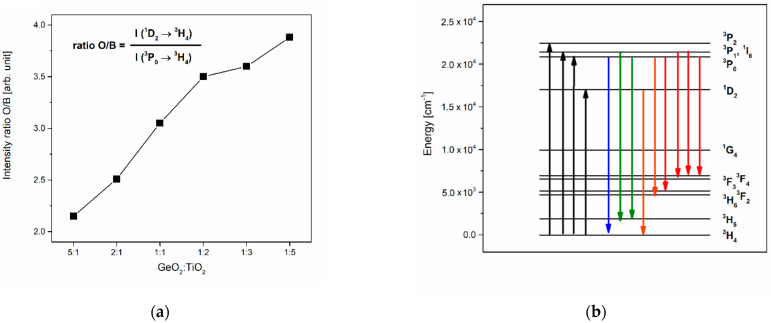
(**a**) The integrated luminescence intensities of orange and blue lines due to main ^1^D_2_ → ^3^H_4_ and ^3^P_0_ → ^3^H_4_ transitions and (**b**) energy level scheme for Pr^3+^ in TiO_2_-modified germanate glasses.

Figure 4 shows the luminescence spectra of glasses containing Pr^3+^ ions recorded under direct excitation by the 450 nm line (^3^P_2_ state). Independently of the GeO_2_:TiO_2_ molar ratio, several luminescence bands were observed, and it should be noticed that the studied systems exhibit blue, green, and intense orange-red emissions. The registered blue and green emission bands at around 484 nm and 526 nm, 540 nm correspond to ^3^P_0_ → ^3^H_4_ and ^3^P_1_ → ^3^H_5_, ^3^P_0_ → ^3^H_5_ transitions, respectively. The spectral range between 570 nm and 760 nm consists of two broad unresolved luminescence groups of bands as a result of overlapping bands originating with the ^1^D_2_ → ^3^H_4_, ^3^P_0_ → ^3^H_6_, ^3^P_0_ → ^3^F_2_, and ^3^P_1_ → ^3^F_3_, ^3^P_1_ → ^3^F_4_, ^3^P_0_ → ^3^F_4_ transitions of Pr^3+^ ions. Among visible luminescence bands, the most intense are due to the ^1^D_2_ → ^3^H_4_, ^3^P_0_ → ^3^H_6_ (608 nm), ^3^P_0_ → ^3^F_2_ (645 nm), and ^3^P_0_ → ^3^H_4_ (484 nm) transitions of praseodymium ions. Furthermore, the intensity of orange-red luminescence increases with the change in the GeO_2_:TiO_2_ molar ratio (Figure 5a) and the reduction in the phonon energy of the glass host. On the other hand, it was stated that the intensity of the emission band due to ^3^P_0_ → ^3^F_2_ decreased in comparison to the band associated with the ^1^D_2_ → ^3^H_4_ transition of Pr^3+^ ions. In accordance with Mallur and Babu [45], who studied the spectroscopic properties of borate glasses, multiphonon relaxation rates can be negligible between the ^3^P_0_ and ^1^D_2_ states of praseodymium ions. As a result of a possible cross-relaxation (CR) process, the excited state ^1^D_2_ level is more populating.

Moreover, Flizikowski et al. [46] reported that emissions attributed to transitions from the ^1^D_2_ state are more sensitive to the changes in glass composition than the ^3^P_0_ emissions. Our experimental results determined that due to the non-radiative transition among CR channel ^3^P_0_:^3^H_4_ → ^1^D_2_:^3^H_6_, the red emission corresponding to the ^1^D_2_ → ^3^H_4_ transition of Pr^3+^ increases. In contrast, emission originating with the transition from the ^3^P_0_ state decreases for glass systems with higher content of TiO_2_. Therefore, it could be stated that the presence of titanium (IV) oxide in the germanate host raises the cross-relaxation processes between Pr^3+^ ions. According to Naresh and Ham [47], the relation between the intensity of orange (^1^D_2_ → ^3^H_4_) and blue (^3^P_0_ → ^3^H_4_) emission, referred to as the orange-to-blue ratio (O/B), depends on glass composition and Pr^3+^ ion concentration. Taking into account the ratio value between the intensity emission at 608 nm and 484 nm, the results demonstrate that the change of glass modifier concentration (TiO_2_) also causes an increase in orange intensity and a decrease in blue emission (Figure 5a). Figure 5b presents the energy level diagram with all visible transitions under the excitation of Pr^3+^ ions in TiO_2_-modified germanate glasses at 450 nm.

**Figure 6:**
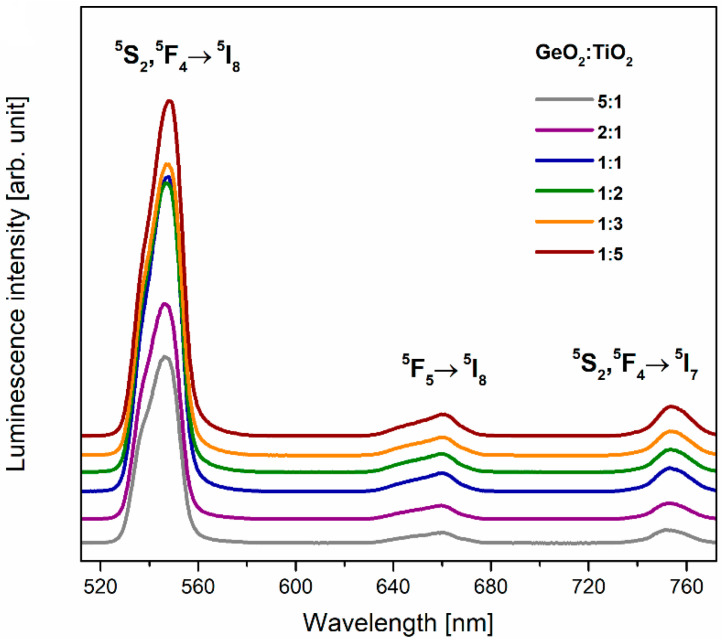
Luminescence spectra for Ho^3+^ ions in TiO_2_-modified germanate glasses.

**Figure 7:**
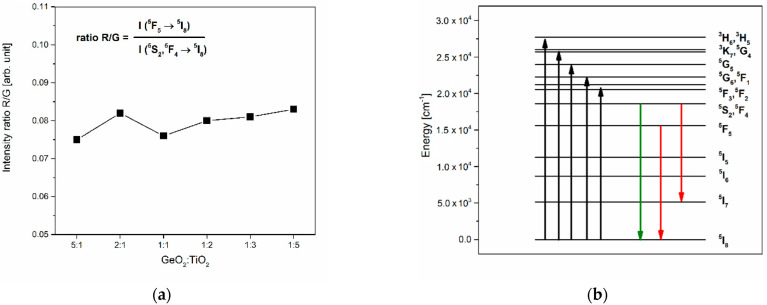
(**a**) The integrated emission intensities of red and green lines due to main ^5^F_5_ → ^5^I_8_ and ^5^S_2_, ^5^F_4_ → ^5^I_8_ transitions and (**b**) energy level scheme for Ho^3+^ in TiO_2_-modified germanate glasses.

Based on recorded excitation spectra for systems doped with Ho^3+^ ions, the 450 nm wavelength was chosen in order to register the registration of luminescence spectra in the function of the GeO_2_:TiO_2_ molar ratio for the studied glass systems (Figure 6). Independently of the chemical composition, the glass systems exhibit green and red emissions, and bands with maxima at around 547, 660, and 752 nm were noticed on the spectra. Furthermore, the most intense band, among emission bands, corresponds to ^5^S_2_, ^5^F_4_ → ^5^I_8_ (green emission) transitions of holmium ions. In agreement with studies for phosphate glasses doped with Ho^3+^ ions [48] and antimony–germanate optical glass fiber co-doped with Yb^3+^/Ho^3+^ [49], the crucial spectroscopic parameter determined based on emission spectra is the R/G ratio. This factor is a relation between the intensity of red (^5^F_5_ → ^5^I_8_) and green (^5^S_2_, ^5^F_4_ → ^5^I_8_) luminescence of holmium ions. Contrary to the spectroscopic properties of studied glasses doped with Pr^3+^ ions, our results for glass systems doped with Ho^3+^ show that the red-to-green ratio is independent of the GeO_2_:TiO_2_ molar ratio (Figure 7a). However, the intensity of both the green (547 nm) and red (660 and 752 nm) emission increase with a change in the TiO_2_-modified germanate glass composition. From this point of view, it can be stated the spectroscopic consequence of the reduction in phonon energy due to a higher TiO_2_ concentration is the achievement of the efficient green luminescence of Ho^3+^ ions. Figure 7b shows the energy level diagram of holmium ions in the studied glass systems.

**Figure 8:**
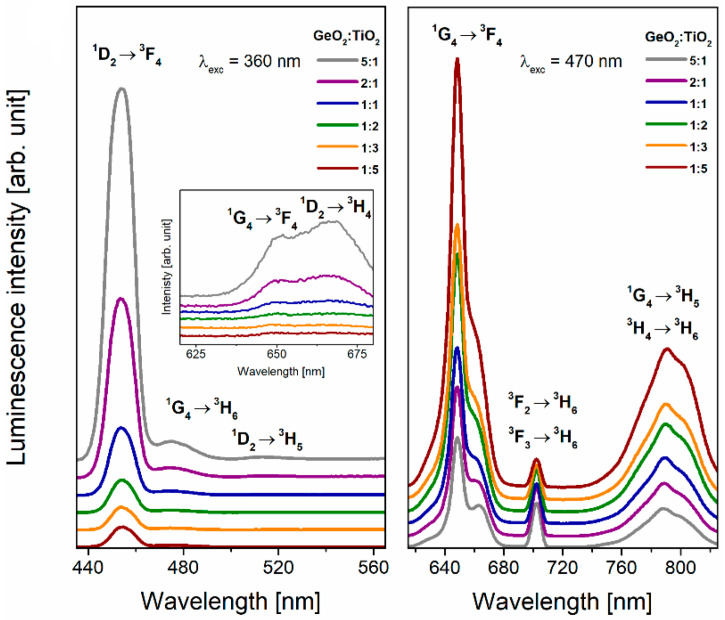
Luminescence spectra for Tm^3+^ in TiO_2_-modified germanate glasses. The inset presents luminescence bands registered in spectral range between 620 and 680 nm.

**Figure 9:**
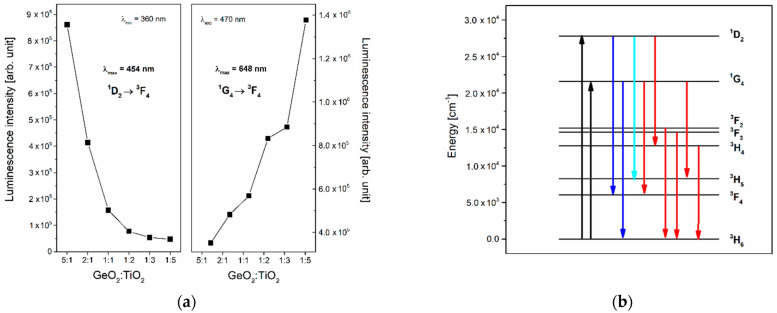
(**a**) The luminescence intensities of blue and red lines due to main ^1^D_2_ → ^3^F_4_ and ^1^G_4_ → ^3^F_4_ transitions and (**b**) energy level scheme for Tm^3+^ ions in TiO_2_-modified germanate glasses.

Next, the emission spectra of Tm^3+^-doped TiO_2_-modified germanate glasses were registered under direct excitation of the ^1^D_2_ state (λ_exc_ = 359 nm) and ^1^G_4_ state (λ_exc_ = 470 nm) (Figure 8). It is worth noting that the studied systems emit blue and red radiations regardless of the GeO_2_:TiO_2_ molar ratio. The luminescence bands due to the transition from the excited states ^1^D_2_, ^1^G_4_, ^3^F_2_, ^3^F_3_, and ^3^H_4_ to lower-lying states of thulium ions were observed for all the studied glass systems. Additionally, it should be noticed that the blue emission band with a maximum at 454 nm is the most intense in comparison with other bands registered under excitation at λ_exc_ = 359 nm (Figure 9a). However, the relatively strong red emission due to the ^1^G_4_ → ^3^F_4_ transition of Tm^3+^ ions was observed when the glass samples were excited by 470 nm. The influence of excitation wavelengths on the intensities of emission corresponding to transitions from the excited states of Tm^3+^ ions is well known and investigated, among other things, by Piramidowicz et al. for fluorozirconate glasses [50]. Analysis of our results indicates that the shape of the emission band (^1^G_4_ → ^3^F_4_) changed with excitation wavelengths and proves the overlapping of two luminescence bands corresponding to the ^1^D_2_ → ^3^H_4_ and ^1^G_4_ → ^3^F_4_ transition of thulium ions. The change of luminescence intensity looks similar to the one observed on the excitation spectra. Nevertheless, a different relationship was shown in the case of a band with a maximum at 702 nm (Figure 8). This emission band was also registered for Yb^3+^/Tm^3+^/Gd^3+^-doped NaYF_4_ [51] and SrYbF_5_ co-doped with Er^3+^/Tm^3+^ ions [52]. Based on the energy level scheme, the observed band can be assigned to ^3^F_2_, ^3^F_3_ → ^3^H_6_ transitions of Tm^3+^ ions. It should be pointed out that a population of the lower-lying states ^3^F_2_ and ^3^F_3_ is due to the cross-relaxation process accompanied by direct pumping of the excited state ^1^G_4_ [53]. Figure 9b presents the energy level diagram with excitation and emission transitions for Tm^3+^ ions in TiO_2_-modified germanate glasses.

### 3.3. CIE Chromaticity Coordinates (x, y) Analysis

Germanate-based glasses singly doped with praseodymium, holmium, and thulium ions can be promising matrices for optoelectronics in RGB technology due to visible emissions. Generally, these rare earth ions are incorporated into glass host matrices to produce systems emitting three primary colors of light: red (Pr^3+^), green (Ho^3+^), and blue (Tm^3+^). The concentration of glass components is a crucial factor of intensity and color emission associated with characteristic transitions of rare earth ions. In order to evaluate the influence of glass composition on color visible luminescence, a CIE diagram and chromaticity coordinates (x, y) are usually employed. These important spectroscopic parameters are regulated by the Commission Internationale de l’Éclairage, defined in 1931, and they express all colors by using the three primary colors of X, Y, and Z. Therefore, the chromaticity coordinates for the studied germanate-based glass systems modified by TiO_2_ were calculated from luminescence spectra and analyzed in detail.

**Figure 10:**
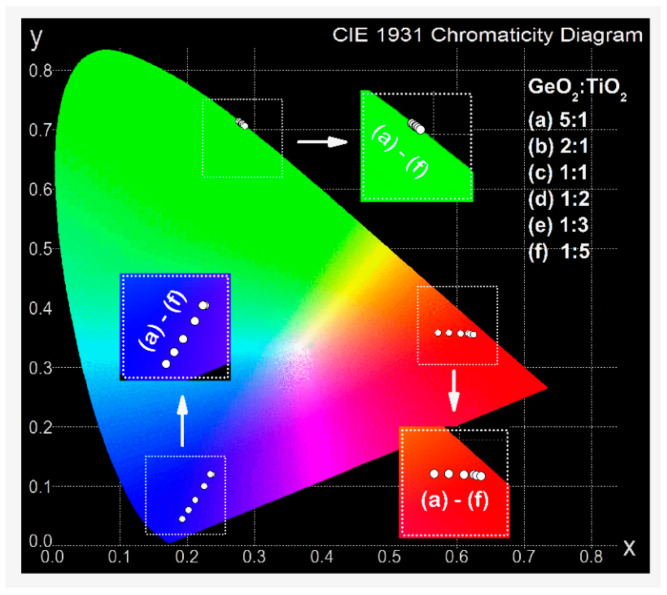
The influence of GeO_2_:TiO_2_ molar ratio on CIE chromaticity coordinates for glasses doped with praseodymium, holmium, and thulium ions.

Figure 10 shows the CIE diagram with marked coordinates (x, y) for systems doped with Pr^3+^, Ho^3+^, and Tm^3+^ ions. The presented results indicate that luminescence color depends on the GeO_2_:TiO_2_ molar ratio in the glass composition. The studied germanate-based glass systems containing Pr^3+^ ions emit red luminescence and chromaticity coordinates evaluated for these glasses shifts to a purer red color when the content of TiO_2_ is changed from 10 mol% to 50 mol%. On the other hand, the x and y coordinates of the glasses doped with Ho^3+^ ions are similar and, regardless of TiO_2_ concentration, point to the green region of the CIE chromaticity diagram. Previously systematic spectroscopic studies confirm that systems containing Ho^3+^ ions are promising candidates for green light emitters [54,55]. Based on the CIE diagram, it was found that all studied glasses doped with Tm^3+^ ions exhibit blue emission, similar to borotellurite, borosilicate [56], as well as phosphate [57] glass systems. Analysis of the CIE chromaticity coordinates calculated for systems doped with Tm^3+^ ions in the function of GeO_2_:TiO_2_ molar ratio demonstrates the opposite tendency in comparison with results obtained for glasses doped with Pr^3+^ ions. The values of coordinates shift to the perfect white light (x = 0.333, y = 0.333) when the concentration of glass component TiO_2_ increases.

Considering the above results for all studied glasses, it can be concluded that TiO_2_ as a glass modifier significantly influences the spectroscopic properties of rare earth ions in germanate glass systems. Therefore, a suitable titanium dioxide concentration allows for receiving optical materials with improved luminescence properties. Titanium dioxide is an important modifier of the germanate matrix, which, depending on the concentration, type of optically active dopant, and excitation wavelength, contributes significantly to the improvement of critical spectroscopic parameters of systems such as emission intensity or CIE chromaticity coordinates. We suggest that the results for TiO_2_-modified germanate glasses doped with Pr^3+^, Ho^3+^, and Tm^3+^ ions are attractive for visible emission, giving an essential contribution to the development of luminescent glasses and celebrating the year 2022 as the International Year of Glass, IYOG [58].

## 4. Conclusions

In summary, the influence of the oxide glass modifier TiO_2_ on the spectroscopic properties of germanate-based systems singly doped with rare earth ions (Pr^3+^, Ho^3+^, and Tm^3+^) was investigated. Independently of the GeO_2_:TiO_2_ molar ratio, intense red (608 nm), green (547 nm), and blue (454 nm) emissions were observed for glasses doped with Pr^3+^, Ho^3+^, and Tm^3+^ ions, respectively. It was demonstrated that the concentration of titanium oxide significantly increases the emission intensity of glass systems containing Pr^3+^ and Ho^3+^ ions. However, blue emission decreases with increasing content of TiO_2_ in germanate-based glasses doped with Tm^3+^ ions. Additionally, it can be concluded that the visible radiative transitions of Pr^3+^, Ho^3+^, and Tm^3+^ ions are greatly dependent on the reduction in the phonon energy of the glass host modified by TiO_2_. Based on CIE chromaticity coordinates (x, y) analysis, it has been proven that the change of GeO_2_:TiO_2_ molar ratio causes significant and opposite color modification for the studied systems doped with Pr^3+^ and Tm^3+^ ions. The addition of TiO_2_ to system compositions doped with Tm^3+^ ions shifts the (x, y) to the center of the CIE diagram. In contrast, chromaticity coordinates evaluated for glasses containing Pr^3+^ ions move to a purer red color. It was confirmed that the spectroscopic properties of germanate-based glasses strongly depend on TiO_2_ content. Our results suggest the applicability of TiO_2_-modified germanate glasses as novel visible photoluminescent materials in RGB technology and photonic devices.

## Data Availability

Not applicable.
